# Cell surface GRP78: A potential marker of good prognosis and response to chemotherapy in breast cancer

**DOI:** 10.3892/ol.2015.3579

**Published:** 2015-08-06

**Authors:** RINAT YERUSHALMI, ANNAT RAITER, KAREN NALBANDYAN, BRITTA HARDY

**Affiliations:** 1 Davidoff Cancer Center, Rabin Medical Center, Beilinson Campus, Petah Tikva 49100, Israel; 2 Sackler School of Medicine, Tel Aviv University, Tel Aviv 6997801, Israel; 3 Felsenstein Medical Research Center, Rabin Medical Center, Beilinson Campus, Petah Tikva 49100, Israel; 4 Department of Pathology, Rabin Medical Center, Beilinson Campus, Petah Tikva 49100, Israel

**Keywords:** breast cancer, 78-kDa glucose-regulated protein, neoadjuvant treatment, prognostic marker, predictive marker

## Abstract

The 78-kDa glucose-regulated protein (GRP78) is a stress induced heat shock protein which, under limiting conditions, functions as a cell surface signaling receptor. Tumor cells are considered to be subjected to a physiologically stressful microenvironment due to their excessive growth. The role of GRP78 in tumor survival has been of notable interest. The present study aimed to assess the potential prognostic and predictive value of cell surface GRP78 expression in breast cancer tumor cells. Cell surface and cytoplasmic expression of GRP78 was examined by immunohistochemical staining of GRP78 in breast cancer archival paraffin-embedded tumor specimens. The cohort studied included breast cancer patients with operable T1,2, estrogen receptor-positive, node-negative cancer who were assessed using the Oncotype DX gene profile, as well as patients with locally advanced disease prior to and following neoadjuvant systemic treatment. GRP78 values were compared between the 2 groups, and prior to and following systemic treatment. Association analyses between GRP78 expression and prognostic markers were also performed. Cox regression analysis was used to examine the impact of these variables on disease-free survival (DFS). No differences in cytoplasmic GRP78 expression were observed. By contrast, the rates of cell surface GRP78 expression were 74.1% in the early stage operable patients, 36% in neoadjuvant systemic treatment patients prior to treatment and 62.5% in patients following systemic treatment (P<0.039). Positive cell surface GRP78 expression was associated with increased expression of the progesterone receptor (P=0.024), p53 expression (P=0.022) and improved DFS (P=0.047). In the case of GRP78 positivity, a trend for a superior response to chemotherapy was observed (P=0.19). The results of the present study indicated that cell surface GRP78 may be used as a marker for good prognosis in breast cancer and a potential marker for response to chemotherapy.

## Introduction

Breast cancer accounts for ~20% of all malignancies in women. Its various subtypes and behaviors have been well documented in recent decades (1). Treatments and outcomes have markedly improved with the introduction of efficient screening programs and the use of novel adjuvant hormonal therapy, chemotherapy and biological agents (2,3). Nevertheless, not all cases are curable. The need to improve understanding of the mechanisms underlying the disease and to provide the most appropriate therapy has prompted researchers to attempt to identify novel prognostic and predictive tumor markers that may serve as targets for future treatments. One such predictive marker is the 78-kDa glucose-regulated protein (GRP78) protein. GRP78 is a key regulator of the unfolded protein response mechanism that underlies endoplasmic reticulum stress and protects cells against apoptosis (4). A number of studies have demonstrated that during endoplasmic reticulum stress, cells overexpress GRP78, inducing its translocation to the cell surface (5–7). Cell surface GRP78 has been identified in various types of cancer, including prostate (8), gastric (9) and ovarian cancer (10), as well as melanoma (11) and astrocytoma (12). Numerous previous studies have identified a correlation between high GRP78 levels and high pathological grade, recurrence and poor survival in various malignancies (9–13); however, other studies have reported the opposite (14,15).

Preclinical studies have indicated that high levels of GRP78 protein may predict resistance to chemotherapy (doxorubucin) or, by contrast, response to a specific type of chemotherapy (taxane) (7,16,17).

The aim of the present study was to determine the impact of GRP78 expression in early and locally advanced breast cancer and to analyze cell surface GRP78 expression compared with traditional prognostic and predictive parameters, as well as Oncotype DX, a validated predictive gene profile test (18).

## Materials and methods

### 

#### Patients and design

The present study included patients with American Joint Committee on Cancer stage I–III (19) breast cancer who were referred to Rabin Medical Center (RMC), Beilinson Campus (Petah Tikva, Israel) with sufficient residual tumor for GRP78 staining. All patients were diagnosed and treated between 2005 and 2012. From this cohort, two patient groups were assigned: Group 1, patients with operable T1,2, node-negative cancer who were assessed with the Oncotype DX gene profile in addition to standard assessment by the local pathologist; group 2, patients who mainly possessed locally advanced tumors, who were assigned to receive neoadjuvant systemic treatment in addition to surgery. Only patients who underwent their initial biopsy study and final surgery at the RMC were included.

The study was approved by the Institutional Review Board of the RMC, in accordance with the Helsinki Declaration of 1975 (20).

#### Data collection

Data on patient and tumor characteristics, including age, stage, grade, estrogen receptor (ER) and progesterone receptor (PR), human epidermal growth factor 2 receptor (HER2), Ki-67 status and p53 protein expression were collected. For group 1 (early breast cancer), the Oncotype DX score was also recorded. For group 2 (locally advanced cancer), the characteristics of the tissues were recorded prior to and following systemic treatment, in addition to the type of chemotherapy received (± trastuzumab) and the rate of response to treatment. A complete pathological response was defined as no invasive residual tumor in the breast or axillary lymph nodes. An almost complete pathological response was defined as residual invasive disease in the breast, measuring <0.1 cm and no residual lymph-node involvement. Disease-free survival (DFS) was reported for all patients.

Breast cancer subtypes were classified according to a gene-expression-profile-validated immunohistochemistry surrogate panel (21,22) as follows: Luminal A, ER-positive and/or PR-positive, HER2-negative and Ki-67 <14%; luminal B, ER-positive and/or PR-positive, HER2-negative and Ki-67 ≥14%; luminal/HER2, ER-positive and/or PR-positive and HER2-positive regardless of Ki-67 status; HER2-enriched, ER- and PR-negative, HER2-positive; triple-negative, ER-, PR- and HER2-negative.

#### GRP78 staining

Samples were immediately fixed with 4% paraformaldehyde and then embedded in paraffin. The paraffin sections were stored at room temperature. Antigen retrieval was performed using an anti-GRP78 BiP antibody (Rabbit immunoglobulin G; Thermo Fisher Scientific, Waltham, MA, USA), according to the manufacturer's instructions. Briefly, histological sections were deparaffinized with xylene (100%; Sigma-Aldrich, Rehovot, Israel) for 20 min and dehydrated in an ethyl alcohol series (100 and 70%; Finkelman Ltd. Chemicals, Petach Tikva, Israel). Antigen unmasking was performed by heating in citrate buffer (pH 6.0; Sigma-Aldrich) using a Biocare Medical Decloaking Chamber™ (Biocare Medical, LLC, Concord, CA, USA). Following antigen unmasking, the sections were cooled to room temperature, washed with wash buffer (Zytomed Systems, Berlin, Germany), submerged in H_2_O_2_ (3%) for 10 min, washed with tap water and rinsed with wash buffer. The slides were then incubated overnight with 1 µg/ml anti-GRP78 antibody in phosphate-buffered saline (pH 7.5) in a moist chamber at 4°C. The next day, the slides were washed with wash buffer and incubated with horseradish peroxidase-conjugated anti-rabbit secondary antibody (ZytoChem-Plus, Berlin, Germany) for 30 min. This procedure was followed by washing in wash buffer and incubation for 1 min with stable diaminobenzidene solution (Innovex Biosciences, Richmond, CA, USA), prepared according to the manufacturer's instructions. The slides were then washed with distilled water for 5 min, counterstained with Harris hematoxylin (Sigma-Aldrich) and permanently mounted with mount medium. Control staining was performed without the primary antibody for nonspecific staining, and was negative.

#### GRP78 expression score

GRP78 expression was analyzed in 15–20 areas of infiltrative carcinoma cells in whole biopsy sections. Analyses were performed separately for cytoplasmic and cell surface staining at x400 magnification (BX-43 microscope; Olympus America, Inc., Center Valley, PA, USA). Cytoplasmic GRP78 staining was graded on a 4-point scale as follows: 0, none; 1, weak; 2, moderately intense; 3, very intense. Cell surface GRP78 staining was recorded as the percentage of cell surface GRP78-positive tumor cells in the whole slide. Membranous staining of <10% of cells was considered negative, and >10% of cells, positive. All scoring was performed by a single investigator blinded to the findings for other pathological stains and patient outcome. Representative images of the slides were captured with an Olympus DP72 camera (lens, x40; Olympus, Tokyo, Japan) using the Cell A software program, version 3.2 (Olympus Soft Imaging Solutions, Münster, Germany).

#### Determination of known tumor markers

Staining for ER, PR, p53, Ki-67 and HER2 was performed using the Ventana Benchmark XT automated immonostainer (Ventana, Tuscon AZ, USA) with the standard cell conditioner (CC1) protocol for 30 min. Following deparaffinization and the CC1 protocol, ready-to-use ER rabbit monoclonal antibody [anti-ER (6F11) primary antibody; Ventana] was applied for 40-min incubation at 37°C; PR rabbit monoclonal antibody (clone 16; Novocastra, Newcastle, UK) was employed at a 1:100 dilution with 40-min incubation at 37°C; Ki-67 rabbit monoclonal antibody (clone SP6; Thermo Fisher Scientific) was used at a 1:100 dilution for 40 min at 37°C; and ready-to-use PATHWAY HER2 anti-HER2/neu rabbit monoclonal antibody (4B5) (Ventana) was utilized with 32-min incubation at 37°C. For HER2 fluorescence *in situ* hybridization (FISH) assay, the slides were hybridized with probes to locus-specific identifier (LSI) HER2/neu and to centromere 17 using the PathVysion HER-2 DNA Probe kit (Abbott Molecular, Abbott Park, IL, USA) according to the manufacturer's instructions. Slides were counterstained with 4,6-diamidino-2-phenylindole (Sigma-Aldrich), and the stained material was visualized under a BX51 fluorescence microscope (Olympus). The signals were analyzed manually.

The ER and PR staining was scored using a modified version of the H-SCORE method: (1 × percentage of weakly staining nuclei + 2 × percentage of moderately staining nuclei + 3 × percentage of intensely staining nuclei)/100, yielding a range of 0–3 (23).

Ki-67 and p53 were evaluated by the percentage of positively stained nuclei (0–100%). HER2 positivity was defined as an IHC of 3. If IHC equaled 2, an amplification ratio ≥2.0 with FISH, was considered positive.

#### Statistical analysis

The expression of cytoplasmic and cell surface GRP78 was compared between patients with early breast cancer and patients who required neoadjuvant systemic treatment, prior to and following the administration of treatment. For categorical variables, Fisher's exact test or χ^2^ test was used to analyze differences in mean values between groups. For ordinal variables, Spearman's nonparametric correlation coefficient was used. Differences in mean parameters prior to and following treatment were analyzed with the Wilcoxon signed rank test. A Kaplan-Meier plot was created for DFS and Cox regression analysis was performed to examine the impact of the variables on DFS. P<0.05 was considered to indicate a statistically significant difference.

## Results

### 

#### Clinicopathological data

Forty-eight patients with breast cancer were included in the study, 27 with operable early cancer (group 1) and 21 who received neoadjuvant chemotherapy (group 2). In addition, 20/21 patients in group 2 presented with locally advanced tumors.

The operable early cancers consisted of luminal A/B subtypes only: Luminal A, 56% and luminal B, 44%. Twenty patients in this group (74%) had stage I disease and 26% had stage II disease. As per the inclusion criteria, none exhibited lymph node involvement. The tumors in group 2, the neoadjuvant group, consisted of various subtypes: Luminal A, 14%; luminal B, 47%; luminal HER2, 24%; HER2-enriched, 5%; and triple-negative, 10%. Of the 21 patients in this group, 15 (71%) presented with stage III disease, 5 (24%) with stage II and one with stage I (5%). All patients in group 2 received anthracycline- and taxane-based regimens. Trastuzumab was administered to 3/6 patients (50%) with HER2-positive disease. Two patients (10%) reached a pathological complete response and 5 (24%), an almost pathological complete response.

#### No significant differences in cytoplasmic GRP78 were detected between groups

**Figure 1.**
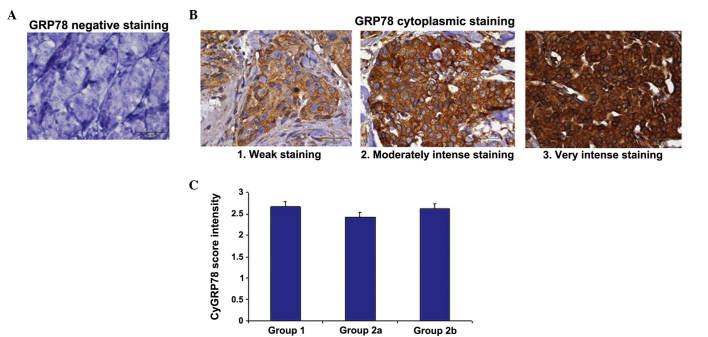
The GRP78 cytoplasmic determination. Representative photomicrographs of immunohistochemical cytoplasmic anti-GRP78 antibody staining in breast tumors. (A) GRP78 cytoplasmic negative staining (score 0). (B) GRP78 weak staining (score 1), moderate intensity (score 2) and intense staining (score 3). Magnification, x400 for all panels. Scale bars, 50 µm. (C) GRP78 score intensity of patients with early operable breast cancer (group 1) compared with patients with locally advanced breast cancer (group 2) prior to (2a) and following (2b) systemic neoadjuvant chemotherapy. No significant differences were observed between the groups. Error bars indicate the standard error of the mean. GRP78, 78-kDa glucose-regulated protein.

The cytoplasmic GRP78 was evaluated in histological sections of the breast cancer patients (Fig. 1). The mean scores for cytoplasmic GRP78 expression were 2.7±0.12 in group 1 (patients with early-stage disease), 2.43±0.11 in group 2 (patients prior to systemic therapy) and 2.65±0.13 in group 2 following systemic therapy. No significant differences were observed between the groups (P>0.5) (Fig. 1C). Fig. 1A depicts the negative control cytoplasmic and cell surface GRP78 staining, while Fig. 1B illustrates the various intensities of GRP78 staining, according to the scores described in the materials and methods section.

#### Cell surface GRP78 expression varies between groups

**Figure 2.**
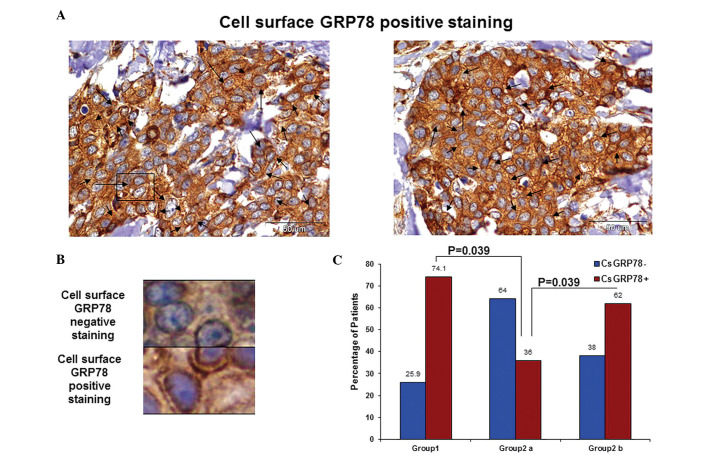
GRP78 cell surface expression varies between groups. (A) Representative images of immunohistochemical cell surface GRP78 staining in breast tumors. Positive staining (>10% of whole slide; arrows). Magnification, x400 for all panels. Scale bars, 50 µm. (B) Representative images demonstrating cell surface GRP78 positive and negative staining. Enlargement of the outlined area in (A). (C) Rates of positive cell surface GRP78 expression. Patients with early operable breast cancer (group 1) compared with patients with locally advanced breast cancer (group 2) prior to (2a) and following (2b) systemic neoadjuvant chemotherapy. The difference in positive cell surface GRP78 expression prior to and following chemotherapy was statistically significant, P<0.039. Group 1 vs. group 2b, P=0.32. GRP78, 78-kDa glucose-regulated protein.

Since no significant differences were observed in GRP78 cytoplasmic determination, all further analyses were based on cell surface GRP78 staining only. A representative sample of positive cell surface GRP78 expression is presented in Fig. 2A and the distinction between positive and negative GRP78 staining is demonstrated in Fig. 2B. In group 1, 74.1% of the cells were positive for cell surface GRP78 and 25.9% were negative; while in group 2, the percentage of positive cell surface GRP78 expression was 36% prior to neoadjuvant systemic treatment, which significantly increased to 62.5% following treatment (P=0.039) (Fig. 2C). Group 1, which included patients with ER-positive disease but no lymph node involvement, demonstrated the highest percentage of patients with cell surface GRP78 expression. Patients in group 2 were significantly less likely to present positive cell surface GRP78 expression prior to systemic treatment compared with afterwards.

The results obtained for cell surface GRP78 expression in group 1 (the luminal, node negative group members who were referred to up-front surgery) were compared by χ^2^ tests to the post neoadjuvant-treated patients (group 2), and no significant differences were observed (P=0.32). By contrast, a significant difference was observed between group 1 and the pre-chemotherapy group 2 (P=0.039).

#### Cell surface GRP78 expression is correlated with PR staining

Table I summarizes the results obtained for the whole cohort. No significant differences were observed between cell surface GRP78 expression and age, tumor size, grade, ER, Ki167 and Oncotype DX score.

A direct correlation between GRP78 expression and the level of PR staining was observed. GRP78 expression was observed in 44.8% of samples with a higher PR score (PR ≥1) and in 10.52% of samples with a lower PR score (PR <1). Positive staining for cell surface GRP78 was therefore more likely to be significantly associated with a higher PR score than with negative staining (P=0.021).

Positive cell surface GRP78 was detected in 61% of the samples with higher p53 protein expression as compared with 39% of the samples with lower p53 protein expression. In this experiment, a higher level of positive cell surface GRP78 correlated significantly with a higher level of p53 expression (P=0.022).

#### Cell surface GRP78 expression is associated with improved DFS

**Figure 3.**
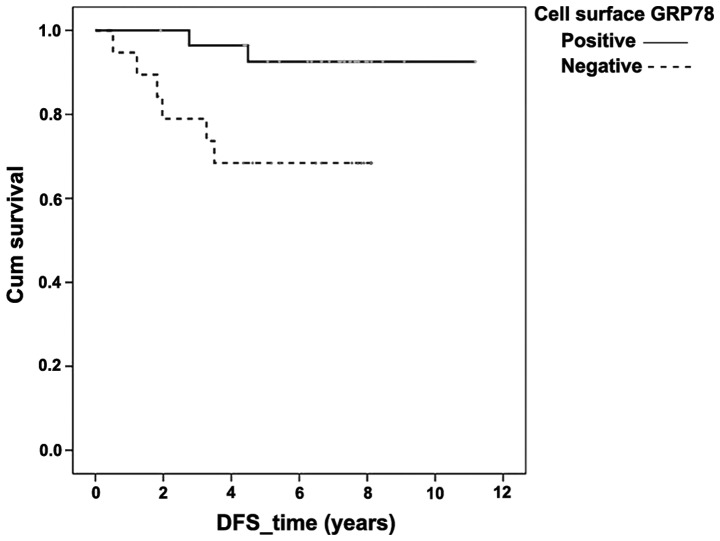
DFS is improved in cell surface GRP78-positive cases. Kaplan-Meier for DFS depending on cell surface GRP78. Positive cell surface GRP78 expression was correlated with improved DFS, P=0.047. DFS, disease-free survival; GRP78, 78-kDa glucose-regulated protein; cum, cumulative.

ER positivity was associated with an improved DFS based on both univariate and multivariate analyses at P=0.004 and P=0.047, respectively (data not shown). Positive cell surface GRP78 expression was also associated with an improved DFS (Fig. 3), P=0.047 in univariate analysis, but demonstrated only a trend in the same direction in multivariate analysis (P=0.070).

#### Association between cell surface GRP78 expression status and predictive parameters

A trend towards an inverse association between cell surface GRP78 expression and an Oncotype DX score was observed, which is predictive of beneficial adjuvant chemotherapy. Sixty-five percent of patients with high expression of cell surface GRP78 (15/27) had a low Oncotype DX score, while 71.4% (19/27) of patients with a high Oncotype DX score demonstrated low expression of cell surface GRP78. Cell surface GRP78 positivity was therefore associated with a lower gene profile score (P=0.185).

In the patients of the neoadjuvant group (group 2), where all tumors except one were locally advanced, a complete or almost complete pathological response was less likely to be associated with GRP78 positivity prior to systemic treatment (P=0.195). Only 25% of the patients with positive cell surface GRP78 expression achieved a complete or almost complete pathological response, compared with 50% of the patients negative for GRP78 expression.

## Discussion

The present study demonstrated that cell surface GRP78 expression may serve as a novel prognostic and predictive marker in breast cancer, to improve the estimation of the recurrence risk and to predict the benefits of systemic treatment. Good prognostic and predictive markers are critical in early and locally advanced breast cancer since the aim of treatment is to cure with minimal toxicity.

One of the challenges of investigating a novel tumor marker is to establish a valid, reproducible scoring method. The scoring method in the present study was based on that of previous publications (13,17,24).

In the present study, negative cell surface GRP78 expression was significantly associated with locally advanced disease, in contrast to previous studies in which positive GRP78 expression was associated with an aggressive phenotype and poor prognosis (24–26). However, in those earlier studies there was no clear distinction between cytoplasmic and cell surface GRP78 expression. To the best of our knowledge, the present study was the first to differentiate between cytoplasmic and cell surface GRP78 expression in breast cancer patients. The present results are supported by those of an earlier study (27) in which cell surface GRP78-positive tumor cells separated by magnetic beads were characterized and their reduced growth and metastatic potential was demonstrated.

The extensive analyses of the present study are consistent with the finding that cell surface GRP78 expression is a good prognostic factor (14,15). The most notable result was the observation that DFS was significantly improved in cases which were positive, as opposed to negative, for cell surface GRP78, as depicted in the Kaplan-Meier graph. These findings were supported by correlational analysis, which demonstrated an association between positive cell surface GRP78 expression and high PR expression, a known marker for good prognosis (28).

An additional correlation was observed between positive cell surface GRP78 expression and high expression of p53 protein, which has been demonstrated to be associated with poor prognosis in breast cancer patients (29). However, studies have revealed that the p53 levels observed by immunohistochemical staining may be misleading as a prognostic factor, since its significance depends on the breast cancer subtype and may be influenced by the type of p53 mutation (30). Therefore, this specific finding requires further investigation.

A preclinical study reported that high GRP78 expression has a predictive value for resistance to doxorubicin (17), although this finding was not consistent in all studies (24). These studies indicated benefits of the use of taxanes in breast cancer, while others have demonstrated that GRP78-positive tumors may be specifically resistant to topoisomerase inhibitors (16,31–33).

At present, guidelines for systemic adjuvant chemotherapy incorporate the use of expensive gene profiling for prognostic and predictive purposes (34–36). In the clinic, patients with node-negative breast cancer who are candidates for adjuvant chemotherapy are routinely offered gene profiling; those with a high score are considered at high risk of recurrence but may benefit from systemic chemotherapy. Since this has become the standard of care, the correlation between the novel tumor marker GPR78 and a popular gene set, the Oncotype DX, was studied. The results demonstrated that 65% of the patients with positive cell surface GRP78 expression had a low Oncotype DX score. Translated into clinical practice, this result indicates that measuring GRP78 expression may aid the identification of a subgroup of patients with a favorable prognosis, who will not benefit from adjuvant (prophylactic) chemotherapy.

Gene profiles, including as Oncotype DX, however, do not yet serve a role in the decision-making process for systemic neoadjuvant chemotherapy. In the present study, all patients in the neoadjuvant subgroup received anthracycline- and taxane-based regimens. A trend towards an improved pathological response to treatment was noted in tumors with low levels of cell surface GRP78 expression. These results are in line with the well-established finding that although aggressive breast cancer tumors indicate poor patient prognosis, they respond better to chemotherapy (37,38).

At the completion of the neoadjuvant systemic therapy, the residual tumor was significantly more likely to exhibit positive cell surface GRP78 expression, compared with the pre-treatment tissue. This finding may be attributed to the fact that chemotherapy treatment activated the endoplasmic reticulum stress response, inducing the unfolding protein response key protein GRP78 and specifically its cell surface expression. This effect has previously been demonstrated in breast cancer cell lines (39). These cells may also be less proliferative and metastatic, as was previously demonstrated (27,39). In addition, the residual tumor with high GRP78 expression may represent residual resistant clones that do not respond to chemotherapy. To the best of our knowledge, this is the first study to investigate cell surface GRP78 expression in a neoadjuvant setting.

Despite the limitation of the present study, which is its relatively small sample size, the value of the GRP78 biomarker was highlighted by the various analyses.

In conclusion, literature regarding the prognostic value of high/low levels of cell surface GRP78 expression in malignancies remains controversial. However, the present study indicated that cell surface GRP78 positivity was an indicator of a good prognosis and may serve as a marker for potential benefit from chemotherapy in breast cancer.

## Figures and Tables

**Table I.** Cell surface GRP78 expression correlation with breast cancer prognostic parameters.

Parameter	Cell surface GRP78	n	Mean ± SD	P-value
Age	N	19	57.94±14.1	0.97
	P	29	57.79±10.7	
Tumor size	N	7	1.77±0.53	0.79
	P	20	1.70±0.63	
Grade	N	16	2.25±0.44	0.84
	P	28	2.28±0.59	
ER	N	19	2.02±0.99	0.99
	P	29	2.02±0.83	
PR	N	19	0.35±0.57	0.021
	P	29	0.97±1.03	
p53	N	18	1.61±2.59	0.022
	P	29	16.17±25.8	
Ki-67	N	18	31.11±20.54	0.31
	P	29	24.31±22.65	
Oncotype DX score	N	7	28.57±5.5	0.75
	P	20	26.30±18.18	

GRP78, 78-kDa glucose-regulated protein; PR, progesterone receptor; SD, standard deviation; ER, estrogen receptor; N, negative; P, positive.
